# A novel mutation of *HOXA11* in a patient with septate uterus

**DOI:** 10.1186/s13023-017-0727-9

**Published:** 2017-12-11

**Authors:** Ying Zhu, Zhi Cheng, Jing Wang, Beihong Liu, Longfei Cheng, Beili Chen, Yunxia Cao, Binbin Wang

**Affiliations:** 1 Reproductive Medicine Center, The First Affiliated Hospital, Anhui Medical University, Hefei, 230032 People’s Republic of China; 2 School of Basic Medical Sciences, Chongqing Medical University, Chongqing, 400016 People’s Republic of China; 3 National Research Institute for Family Planning, Beijing, 100081 People’s Republic of China; 4 Center for Genetics, National Research Institute for Family Planning, 12, Dahuisi Road, Haidian, Beijing, 100081 China

**Keywords:** Mutation, *HOXA11*, Septate uterus patients

## Abstract

**Background:**

The etiology of Müllerian duct anomalies (MDAs) is poorly understood at present. The *HOXA11* gene is crucial for the development of the Müllerian duct. The objective of this study is to report a unique case of MDAs with a novel mutation in *HOXA11*.

**Results:**

We identified a potential disease-causing mutation (p. E255K) in a patient with a septate uterus. The mutation was not detected in 169 control subjects or listed in any databases of variations. Bioinformatic predictions and functional studies showed that the mutation reduces the DNA binding affinity and disrupts transactivation ability of HOXA11.

**Conclusion:**

In conclusion, this is the first report to describe a *HOXA11* mutation in Chinese women with MDAs. The results demonstrated that mutation in *HOXA11* can contribute to the etiology of MDAs, especially the septate uterus, but might not be a common cause.

**Electronic supplementary material:**

The online version of this article (10.1186/s13023-017-0727-9) contains supplementary material, which is available to authorized users.

## Background

During the embryonic development in female eutherian mammals, two paired Müllerian ducts ultimately develop into the oviducts, uterus, cervix and upper portion of the vagina, which constitute most of the female reproductive tract [1]. Interruptions or disturbances at different stages of this process can result in Müllerian duct anomalies (MDAs). The anomalies are estimated to affect about 5.5% of the general population and are often associated with other organ anomalies, such as renal agenesis and skeletal anomalies [2–4]. MDAs can also impair the sexual and reproductive function of women to different extents [5].

The etiology of MDAs remains largely unknown at present, and seems to be multifactorial. Previous studies showed that environmental and iatrogenic factors, such as exposure to ionizing radiation, viral infections and the use of medications such as methotrexate or diethylstilbestrol (DES), might underlie the occurrence of such malformations [6]. Furthermore, familial aggregation of MDAs was observed among first-degree relatives, which strongly implicates linked genetic factors [7]. Studies in animal models have suggested candidate genes potentially involved in human MDAs. However, most of these candidate genes were failed to be proved pathogenic [8].

*HOX* genes encode evolutionarily conserved transcription factors and exist in almost all metazoans [9]. Humans have 39 *HOX* genes arranged in four separate clusters, termed *HOXA*, *B*, *C* and *D* [10]. Each of the *HOX* genes contains a homeobox sequence encoding a homeodomain, a highly conserved DNA-binding motif. *HOX* genes act as key regulatory factors in embryonic morphogenesis and cell differentiation [11, 12]. Mutations in *HOX* genes have been shown to cause synpolydactyly and hand–foot–genital syndrome [13, 14].

The *HOXA11* gene located in the *HOXA* gene cluster is crucial for the development of the Müllerian duct. It is expressed in the mouse Müllerian duct during embryonic development and subsequently expressed in the lower uterus and uterine cervix in neonates [15]. In *Hoxa11* null mice, the uterus is smaller than normal and shows some anatomical similarities with the more anterior oviducts [16, 17]. Furthermore, alterations in the expression of *Hoxa11* caused by DES exposure resulted in uterine anomalies [18]. Thus, *HOXA11* may contribute to the etiology of MDAs. Here we performed genetic analyses of *HOXA11* in Chinese patients with MDAs.

## Methods

### Subjects

A cohort of 163 Chinese women with MDAs was recruited in our study. Their clinical diagnoses were based on physical examination, ultrasonographic investigations, hysteroscopy and laparoscopy. A group of 169 unrelated healthy women were also screened as controls. Informed written consent was obtained from all individuals. The study protocol was in accordance with the tenets of the Declaration of Helsinki and was approved by the Anhui Medical University ethics committee.

### Genetic analysis

Genomic DNA was extracted from peripheral blood samples using standard methods. The two exons and exon–intron boundaries of the *HOXA11* gene were amplified by polymerase chain reaction (PCR) using two pairs of gene specific primers (Additional file 1: Table S1). The PCR products were sequenced on an ABI 3730XL DNA sequencer (Applied Biosystems, Foster City, CA, USA) using the BigDye Terminator Cycle Sequencing kit (Applied Biosystems).

To validate the novel mutation discovered in the study, PCR amplifications were repeated three times and the products were sequenced in both directions. The novelty of mutation was verified by consulting the National Center for Biotechnology Information single nucleotide polymorphism database (dbSNP) and 1000 Genome Project database. Conservation analysis was performed by using CLC Main Workbench Software. PMut (http://mmb.pcb.ub.es/PMut/), PolyPhen-2 (http://genetics.bwh.harvard.edu/pph2/) and SIFT (http://sift.jcvi.org/) were used to predict the effects of mutation on the protein function.

### Plasmid construction

The human *HOXA11* open reading frame (ORF) was PCR-amplified by using *HOXA11* cDNA as template. The amplification generated Bam HI and Xba I sites into the 5′ and 3′ ends of the HOXA11 ORF. The PCR product was inserted into pMD18-T simple vector (Takara, Dalian, Liaoning, P. R. China). Site-directed mutagenesis was performed on this template to generate a *HOXA11* sequence harboring the p. E255K mutation. Then the wild-type and mutated *HOXA11* ORF were cloned into pcDNA3.1(+) expression vector (Invitrogen Life Technologies, Carlsbad, CA, USA), respectively. A FOXO1 expression plasmid was created by cloning its ORF into the Bam HI and Xba I sites of the pcDNA3.1(+) vector. For the reporter plasmid, the promoter region of the human *PRL* gene (from −519 to +65) was cloned into the Kpn I and Nhe I sites of pGL3-basic vector (Promega, Madison, WI, USA). All the plasmids were verified by sequencing. The PCR primers used for plasmid construction are shown in Additional file 1: Table S2.

### Electrophoretic mobility shift assay (EMSA)

293FT cells were cultured in Dulbecco’s Modified Eagle Medium supplemented with 10% fetal bovine serum, 100 U/ml penicillin and 0.1 mg/ml streptomycin. For transient transfection, cells were cultured in 10 cm dishes until they reached 70% confluency. 2 μg *HOXA11* wild-type and mutant expression constructs were transfected separately into cells using the calcium phosphate method.

Nuclear extracts of the transfected cells were prepared using a nuclear protein extraction kit (Viagene Biotech, Ningbo, P. R, China). Protein concentration was determined by the BCA method. Decreasing amounts of nuclear protein from each sample were mixed with 1.5 μl 10 × Binding Buffer and 1.5 μl Poly(dI-dC) in a 15 μl reaction volume and were incubated for 20 min at room temperature. Then 0.5 μl of a biotin-labeled OPN5 oligonucleotide probe (5′–TAGTTAATGACATCGTTCATCAG–3′) containing the Hox binding site was added to the samples [19]. Following a further incubation for 20 min at room temperature, the samples were separated by 5.5% nondenaturing polyacrylamide gel electrophoresis at 180 V for 70 min. Then the products were transferred to a binding membrane in 0.5 × TBE at 390 mA for 40 min. To crosslink DNA, the membrane was placed in a UV StrataLinker 1800 (Stratagene, La Jolla, CA, USA) following the manufacturer’s instructions. The membrane was then blocked, exposed to streptavidin–horseradish peroxidase, washed four times, and equilibrated. The bands were visualized by chemiluminescence reaction, and images were captured using the Cool II Imager System (Viagene Biotech, Ningbo, P. R. China).

### Transactivation assay

HeLa cells were cultured in DMEM containing 10% fetal bovine serum. For transient transfection, cells were grown to 70–80% confluency on 24-well plates and transfected using Lipofectamine 2000 (Invitrogen Life Technologies) according to the manufacturer’s instructions.

Aliquots of 150 ng of HOXA11 expression constructs and a pcDNA3.1 empty vector were transfected into the cells with 300 ng of *PRL* reporter plasmid and 150 ng of *FOXO1* expression plasmid, respectively. The pRL-TK plasmid encodes *Renilla* luciferase (5 ng) was also added in each cotransfection to normalize the transfection efficiency. 48 h after transfection, cells were harvested and assayed for luciferase activity using the Dual Luciferase Reporter Assay System (Promega). The experiments were repeated three times in triplicate. Statistical significance was determined using independent-sample *t* tests.

## Results

### Phenotypes of analyzed patients

The clinical phenotypes of all 163 patients are shown in Table 1 according to the vagina/cervix/uterus/adnexa-associated malformation classification [20]. The patients showed a broad spectrum of MDAs. Some patients also had associated malformations of the kidneys, skeletons and hearts.

**Table 1:** Clinical characteristics of all patients with Müllerian duct anomalies

Vagina	Number	Uterus	Number	Cervix	Number
Normal	119	Normal	2	Normal	96
Incomplete septate vagina <50%	7	Arcuate	10	Duplex cervix	34
Complete septate vagina	8	Septate <50% of the uterine cavity	52	Unilateral aplasia	16
				Other	17
Hypoplasia	14	Septate >50% of the uterine cavity	42	Associated Malformation	Number
Complete atresia	15	Bicornate	11	None	154
Adnexa	Number	Hypoplastic uterus	30	Renal system	4
Normal	151	Unilaterally rudimentary or aplastic	16	Skeleton	2
Bilateral gonadal streak	1			Cardiac	1
Other	11			Other	2

### Identification of a *HOXA11* missense mutation and case report

**Fig. 1:**
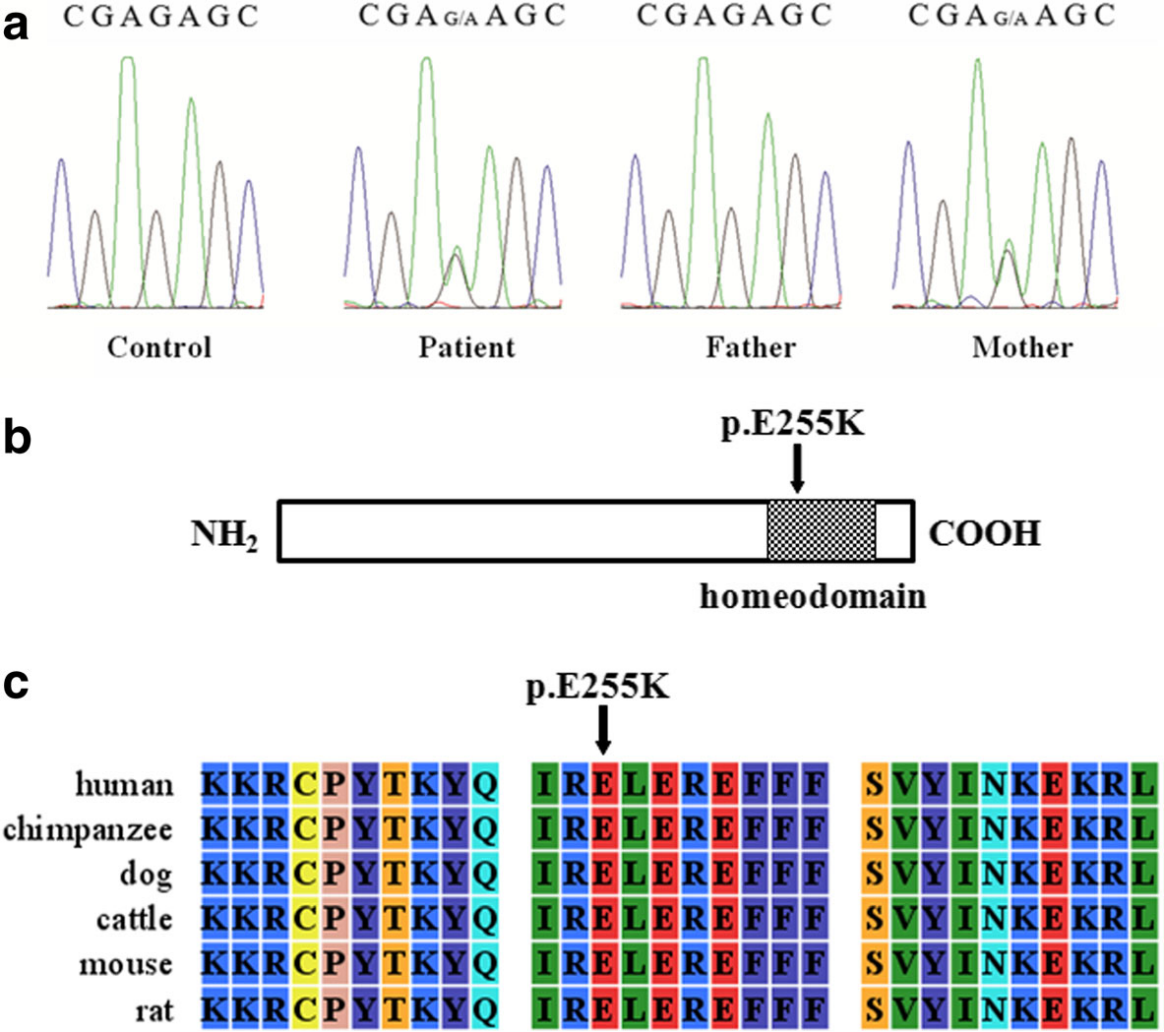
A novel variation of HOX11 identified in a patient with septate uterus. **a** Electropherograms obtained by direct sequencing of PCR products showing the heterozygous G to A substitution at nucleotide 763 of *HOXA11* gene in the patient and the patient’s mother, but not in a control subject or in the patient’s father. **b** The p.E255K mutation situated in the homeodomain of HOXA11. **c** Sequence alignment of HOXA11 protein among different species showing the mutation located in the highly conserved site of the protein

We analyzed the coding region of *HOXA11* gene in 163 patients and 169 healthy controls. Our analysis identified a novel missense mutation- c.763G > A (p. E255K) in a patient with a complete septate uterus, and it was not detected in 169 healthy controls. This mutation was also observed in her mother, who showed a normal uterus (Fig. 1A). Her mother had a history of spontaneous abortion caused by the natural termination of the fetus growth.

The mutation carrier was 36 years old and had spontaneous abortion four times. She had been diagnosed with secondary infertility. Her menstrual cycle was normal. She had normal body mass index (19.9 kg/m^2^) and blood pressure (110/76 mmHg) according to a recent physical examination. Her laboratory tests showed normal levels of total testosterone (0.974 nmol/l), follicle-stimulating hormone (FSH) (9.8 U/l), luteinizing hormone (LH) (5.05 U/l), and estradiol (288.25 pmol/l). Abdominal and pelvic ultrasound examinations and hysteroscopy revealed a septum filling >50% of the uterus. The maximum thickness of her endometrium was only 6.5 mm. The patient did not show any anomalies in the limbs, kidneys, ureters, or central nervous system.

The p. E255K mutation is not reported in dbSNP or the 1000 Genome Project databases. It alters amino acid residues that lie in the homeodomain and is highly conserved among different species (Fig. 1B, C). Furthermore, all three software tools predicted the p. E255K mutation to be possibly damaging. The results of the predictions are shown in Additional file 1: Table S3.

### Functional analyses of *HOXA11* mutation

**Fig. 2:**
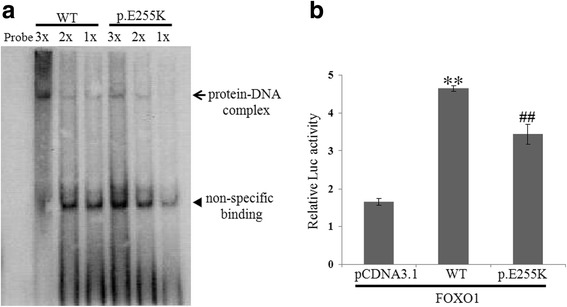
Functional analysis of HOXA11 sequence mutation. **a** Electrophoretic mobility shift assay of nuclear extracts from transfected 293FT cells. 293FT cells were transfected, respectively, with wild type and mutant HOXA11 expression constructs. Decreasing amounts of nuclear extracts were mixed with labeled DNA probes. Protein-DNA complex is indicated by an arrow and a non-specific band is marked with an arrowhead. **b** Transactivation assay in cotransfected HeLa cells. Wild type and mutant HOXA11 expression constructs were cotransfected with *PRL* reporter plasmid, FOXO1 expression plasmid and pRL-TK plasmid. Firefly and Renilla luciferase activities were measured 48 h after transfection. Relative luciferase activity was determined by normalizing the firefly luciferase activity with Renilla luciferase activity. The experiments were repeated three times in triplicate. Representative data shown are expressed as mean ± SD. Statistical significance was determined by the independent-samples *T* test. **, *p* < 0.01 versus pcDNA3.1 empty vecotor. ##, *p* < 0.01 versus wild-type

Because the p.E255K mutation is located in the homeodomain which is considered to relate to DNA binding efficiency we first carried out EMSA using nuclear extracts from 293FT cells transfected with wild-type and mutant expression constructs to investigate whether the mutation would affect the DNA binding properties of *HOXA11* (Fig. 2A). For the wild-type protein, the formation of a protein–DNA complex was as efficient as previously reported. In contrast, the p. E255K mutant showed a reduced capacity to bind DNA, indicating that the mutation greatly reduces the normal DNA-binding affinity of *HOXA11*. Furthermore, we tested the influence of this mutation through a reporter gene transactivation using the *PRL* luciferase reporter. The results are shown in Fig. 2B. When cotransfected with a *FOXO1* expression construct, the wild-type *HOXA11* resulted in a 2.8-fold increase in relative luciferase activity compared with the pcDNA3.1 empty vector. Cotransfection of the p. E255K mutant also enhanced the luciferase activity, but it was decreased by 26% when compared with the wild-type (*p* < 0.001). These results indicate that the p. E255K mutation significantly disrupts the transactivation ability of *HOXA11*.

## Discussion

The normal development of the Müllerian duct involves a complex series of events: elongation, fusion, canalization, and septal resorption. Failure to complete any part of this sequence can lead to MDAs. The manifestations of MDAs vary widely among patients, and uterine anomalies are the most common. Although some genetic factors that regulate the development of the Müllerian duct have been identified, the etiology of MDAs is poorly understood at present.

Here we present the evidence that mutation in *HOXA11* can contribute to the etiology of MDAs, especially the septate uterus. Our study started with a genetic analysis of *HOXA11* gene in Chinese women with MDAs and detected a novel non-synonymous mutation. The mutation was not observed in controls or listed in current variation databases.

The p.E255K mutation is located in the homeodomain of *HOXA11*, which is highly conserved among many different species. It results in a glutamic acid-to-lysine substitution. The glutamic acid is an acid residue with negative charge, while the lysine is a positively charged residue. This substitution might damage the structure of the protein and impair its DNA-binding ability. Bioinformatic predictions and functional analyses both showed that the mutation impairs the function of HOXA11. Therefore, the p. E255K mutation is a potential disease-causing mutation.

There are increasing evidences indicate that HOXA11 plays an essential role in the uterine development and human fertility. In the development of uterus, Hoxa11 promotes proper proliferation and survival of stromal cells. The *Hoxa11* adult mutant uterus displayed little to no stromal tissue and exhibited a reduced diameter [17, 21]. During pregnancy, Hoxa11 was critical for endometrial receptivity and embryonic implantation [22]. Hoxa11 mutated female mice showed high embryo absorption rates because of implantation failure [17]. In another murine model, knockdown of Hoxa11 led to significantly reduced implantation rates [23]. Furthermore, *HOXA11* expression dramatically increased at the time of implantation and remained high in pregnancy [24]. And the reduced expression of *HOXA11* was reported in women with defective implantation [25]. The patient’s endometrium was as thin as only 6.5 mm. She also had spontaneous abortion four times. These phenotypes may owe to the defective uterine development and impaired endometrial receptivity caused by HOXA11 mutation.

It was noteworthy that the patient’s mother also carried the same mutation, but showed normal uterus. The main reason behind this may due to low penetrance. The similar phenomenon has also been reported in previous studies of synpolydactyly, in which some members of the families who carry the *HOXD13* mutation showed normal phenotypes in the hands and feet [26, 27]. Furthermore, the patient’s mother also had a history of spontaneous abortion. It was quite possible that she also had defects in the endometrium as her daughter.

The phenotype of the p. E255K mutation carrier is a complete septate uterus, which results from the failed resorption of the medial septum after the fusion of Müllerian ducts [28]. And our previous research identified a novel mutation of *HOXA10* in a patient with didelphic uterus [29]. Moreover, several nonsense mutations (p.W369X, p.S136X, p.Q196X, and p.Q365X), missense mutations within the homeodomain (p.I368F, p.N372H and p.V375F), and in-frame polyalanine expansions of *HOXA13* gene have been found to cause Müllerian duct fusion defects in patients with hand-foot-genital syndrome [14, 30–32]. Taken together, these results suggest that *HOX* genes might play important roles in the fusion of the Müllerian duct and in the resorption of the uterine septum. Further investigations such as transgenic mice studies are required to elucidate the precise molecular mechanisms.

In the present study, mutation of *HOXA11* was only detected in 0.6% (1/163) of the patients. A previous study that screened *HOXA11* gene in 192 patients with MDAs identified no mutation [33]. These results demonstrated that mutation in *HOXA11* might not be a common cause for MDAs. Other studies regarding *WNT9B*, *PBX1*, *PAX2*, *LHX1*, *HOXA7* and *HOXA9* also got similar results. Mutation screening of these genes had been performed in Chinese patients with MDAs, but no mutation was detected [34–38]. These results suggested that non-genetic factors might contribute to the pathogenesis of MDAs.

## Conclusion

In conclusion, this is the first report to describe a *HOXA11* mutation in Chinese women with MDAs. The results demonstrated that mutation in *HOXA11* can contribute to the etiology of MDAs, especially the septate uterus, but might not be a common cause.

## Additional file

Additional file 1: Table S1. Primers used for PCR. **Table S2** Primers used for site-directed mutagenesis and plasmids construction. **Table S3** Functional significance of HOXA11 mutation by bioinformatic prediction. (DOC 96 kb)

## References

[1] Kobayashi A, Behringer RR (2003). Developmental genetics of the female reproductive tract in mammals. Nat Rev Genet.

[2] Chan YY, Jayaprakasan K, Zamora J, Thornton JG, Raine-Fenning N, Coomarasamy A (2011). The prevalence of congenital uterine anomalies in unselected and high-risk populations: a systematic review. Hum Reprod Update.

[3] Li S, Qayyum A, Coakley FV, Hricak H (2000). Association of renal agenesis and mullerian duct anomalies. J Comput Assist Tomogr.

[4] Golan A, Langer R, Bukovsky I, Caspi E (1989). Congenital anomalies of the mullerian system. Fertil Steril.

[5] Vallerie AM, Breech LL (2010). Update in Mullerian anomalies: diagnosis, management, and outcomes. Current opinion in obstetrics & gynecology.

[6] Ribeiro SC, Tormena RA, Peterson TV, Gonzales Mde O, Serrano PG, Almeida JA (2009). Mullerian duct anomalies: review of current management. Sao Paulo medical journal = Revista paulista de medicina.

[7] Hammoud AO, Gibson M, Peterson CM, Kerber RA, Mineau GP, Hatasaka H (2008). Quantification of the familial contribution to mullerian anomalies. Obstet Gynecol.

[8] Jacquinet A, Millar D, Lehman A (2016). Etiologies of uterine malformations. Am J Med Genet A.

[9] Carroll SB (1995). Homeotic genes and the evolution of arthropods and chordates. Nature.

[10] Scott MPA (1993). Rational nomenclature for vertebrate homeobox (HOX) genes. Nucleic Acids Res.

[11] Krumlauf R (1994). Hox genes in vertebrate development. Cell.

[12] Lawrence HJ, Sauvageau G, Humphries RK, Largman C (1996). The role of HOX homeobox genes in normal and leukemic hematopoiesis. Stem Cells.

[13] Muragaki Y, Mundlos S, Upton J, Olsen BR (1996). Altered growth and branching patterns in synpolydactyly caused by mutations in HOXD13. Science.

[14] Mortlock DP, Innis JW (1997). Mutation of HOXA13 in hand-foot-genital syndrome. Nat Genet.

[15] Taylor HS, Vanden Heuvel GB, Igarashi PA (1997). Conserved Hox axis in the mouse and human female reproductive system: late establishment and persistent adult expression of the Hoxa cluster genes. Biol Reprod.

[16] Hsieh-Li HM, Witte DP, Weinstein M, Branford W, Li H, Small K (1995). Hoxa 11 structure, extensive antisense transcription, and function in male and female fertility. Development.

[17] Gendron RL, Paradis H, Hsieh-Li HM, Lee DW, Potter SS, Markoff E (1997). Abnormal uterine stromal and glandular function associated with maternal reproductive defects in Hoxa-11 null mice. Biol Reprod.

[18] Du H, Taylor HS (2015). The role of Hox genes in female reproductive tract development, adult function, and fertility. Cold Spring Harbor perspectives in medicine.

[19] Li X, Nie S, Chang C, Qiu T, Cao X (2006). Smads oppose Hox transcriptional activities. Exp Cell Res.

[20] Oppelt P, Renner SP, Brucker S, Strissel PL, Strick R, Oppelt PG (2005). The VCUAM (vagina cervix uterus Adnex-associated malformation) classification: a new classification for genital malformations. Fertil Steril.

[21] Wong KH, Wintch HD, Capecchi MR (2004). Hoxa11 regulates stromal cell death and proliferation during neonatal uterine development. Mol Endocrinol.

[22] Taylor HS (2000). The role of HOX genes in human implantation. Hum Reprod Update.

[23] Xu B, Geerts D, Bu Z, Ai J, Jin L, Li Y (2014). Regulation of endometrial receptivity by the highly expressed HOXA9, HOXA11 and HOXD10 HOX-class homeobox genes. Hum Reprod.

[24] Taylor HS, Igarashi P, Olive DL, Arici A (1999). Sex steroids mediate HOXA11 expression in the human peri-implantation endometrium. J Clin Endocrinol Metab.

[25] Taylor HS, Bagot C, Kardana A, Olive D, Arici AHOX (1999). Gene expression is altered in the endometrium of women with endometriosis. Hum Reprod.

[26] Goodman F, Giovannucci-Uzielli ML, Hall C, Reardon W, Winter R, Scambler P (1998). Deletions in HOXD13 segregate with an identical, novel foot malformation in two unrelated families. Am J Hum Genet.

[27] Debeer P, Bacchelli C, Scambler PJ, De Smet L, Fryns JP, Goodman FR (2002). Severe digital abnormalities in a patient heterozygous for both a novel missense mutation in HOXD13 and a polyalanine tract expansion in HOXA13. J Med Genet.

[28] Chandler TM, Machan LS, Cooperberg PL, Harris AC, Chang SD (2009). Mullerian duct anomalies: from diagnosis to intervention. Br J Radiol.

[29] Cheng Z, Zhu Y, Su D, Wang J, Cheng L, Chen B (2011). A novel mutation of HOXA10 in a Chinese woman with a Mullerian duct anomaly. Hum Reprod.

[30] Cao L, Chen C, Leng Y, Yan L, Wang S, Zhang X (2017). A missense mutation of HOXA13 underlies hand-foot-genital syndrome in a Chinese family. J Genet.

[31] Imagawa E, Kayserili H, Nishimura G, Nakashima M, Tsurusaki Y, Saitsu H (2014). Severe manifestations of hand-foot-genital syndrome associated with a novel HOXA13 mutation. Am J Med Genet A.

[32] Goodman FR, Bacchelli C, Brady AF, Brueton LA, Fryns JP, Mortlock DP (2000). Novel HOXA13 mutations and the phenotypic spectrum of hand-foot-genital syndrome. Am J Hum Genet.

[33] Chen X, Li G, Qin Y, Cui Y, You L, Chen ZJ (2014). Mutations in HOXA11 are not responsible for Mullerian duct anomalies in Chinese patients. Reprod BioMed Online.

[34] Tang R, Dang Y, Qin Y, Zou S, Li G, Wang Y (2014). WNT9B in 542 Chinese women with Mullerian duct abnormalities: mutation analysis. Reprod BioMed Online.

[35] Ma J, Qin Y, Liu W, Duan H, Xia M, Chen ZJ (2011). Analysis of PBX1 mutations in 192 Chinese women with Mullerian duct abnormalities. Fertil Steril.

[36] Wang P, Zhao H, Sun M, Li Y, Chen ZJ (2012). PAX2 in 192 Chinese women with Mullerian duct abnormalities: mutation analysis. Reprod BioMed Online.

[37] Xia M, Zhao H, Qin Y, Mu Y, Wang J, Bian Y (2012). LHX1 mutation screening in 96 patients with mullerian duct abnormalities. Fertil Steril.

[38] Chen X, Mu Y, Li C, Li G, Zhao H, Qin Y (2014). Mutation screening of HOXA7 and HOXA9 genes in Chinese women with Mullerian duct abnormalities. Reprod BioMed Online.

