# Supplementary material for: A novel mutation of HOXA11 in a patient with septate uterus
Source: Orphanet J Rare Dis. 2017 Dec 11;12:178. doi: 10.1186/s13023-017-0727-9 (PMC5725892; doi:10.1186/s13023-017-0727-9)
Supplement: Additional file 1: Table S1. — Primers used for PCR. Table S2 Primers used for site-directed mutagenesis and plasmids construction. Table S3 Functional significance of HOXA11 mutation by bioinformatic prediction. (DOC 96 kb) [file 13023_2017_727_MOESM1_ESM.doc]

**Table S**1 Primers used for PCR

| Extron | Primer ID | Sequence |
| --- | --- | --- |
| I | HOXA11-I-forward | 5’-CTGCTAAGGATGGGGATAGAT-3’ |
|  | HOXA11-I-reverse | 5’ -AAAGGGAAAAGGGGAGTTGTT-3’ |
| II | HOXA11-II-forward | 5’-GGGCAGACTTTGACGG-3’ |
|  | HOXA11-II-reverse | 5’ -TCCCAAACCTGTCATTCTAGC-3’ |

**Table S2 Primers used for site-directed** mutagenesis and plasmids construction

|  | Sequences |
| --- | --- |
| Primers used to construct HOXA11 p.A99T variant | Forward: 5’-GGCGCCCAGCACGGCCGGCGT-3’ |
|  | Reverse: 5’-ACGCCGGCCGTGCTGGGCGCC-3’ |
| Primers used to construct HOXA11 p.E255K variant | Forward: 5’-CCTATACCAAGTACCAGATCCGAAAGCTGGAACGGG-3’ |
|  | Reverse: 5’-CCCGTTCCAGCTTTCGGATCTGGTACTTGGTATAGG-3’ |
| Primers used to construct HOXA11 expression plasmid | Forward: 5’-CGCGGATCCATGGATTTTGATGAGCGT-3’ |
|  | Reverse: 5’-CTAGTCTAGATTAGAGGAGTGGATTTGC-3’ |
| Primers used to construct FOXO1 expression plasmid | Forward: 5’-CGCGGATCCATGGCCGAGGCGCCTCA-3’ |
|  | Reverse: 5’-CTAGTCTAGATCAGCCTGACACCCAGCTATG-3’ |
| Primers used to construct *PRL* reporter plasmid | Forward: 5’-CGGGGTACCCTCATCTCCATTATTGACTGC-3’ |
|  | Reverse: 5’-CTAGCTAGCTGTCTCTGTCTTTGAGGG-3’ |

**Table S3 Functional significance of HOXA11 mutation by bioinformatic prediction**

| Mutation | PMut  Prediction | Score | PolyPhen-2  Prediction | Score | SIFT  Prediction | Score |
| --- | --- | --- | --- | --- | --- | --- |
| p.E255K | Pathological | 0.7078 | Probably damaging | 1.000 | Affect protein function | 0.00 |
